# Genotoxic Effect of Lead and Cadmium on Workers at Wastewater Plant in Iraq

**DOI:** 10.1155/2020/9171027

**Published:** 2020-07-25

**Authors:** Salih Ibrahem, Muna Hassan, Qais Ibraheem, Khalid Arif

**Affiliations:** ^1^Department of Biochemistry, College of Medicine, Kirkuk University, Kirkuk, Iraq; ^2^Department of Biochemistry, College of Medicine, Al-Nahrain University, Baghdad, Iraq; ^3^Department of Biochemistry, Faculty of Agricultural Engineering Sciences, Baghdad University, Baghdad, Iraq; ^4^Department of Physiology, College of Medicine, Kirkuk University, Kirkuk, Iraq

## Abstract

Heavy metal poisoning is a worldwide problem that is caused by different human industrial activities such as battery and painting manufacturing and occupational exposure of those working at petrol stations. Wastewater is known to contain higher amounts of heavy metals such as lead (Pd) and cadmium (Cd) and might be sources of exposure for workers at the wastewater treatment plant. However, to our best knowledge, no studies were done to evaluate the level of cadmium and lead in blood of workers at wastewater treatment plants and evaluate the subsequent effect of lead and cadmium on delta-aminolevulinic acid dehydratase (*δ*-ALAD), urinary delta-aminolevulinic acid (U*δ*-ALA), and 8-hydroxy-2′-deoxyguanosine (8-OHdG) as markers of lead and cadmium toxicity. In this case-control study, 79 workers at the Al-Rustumiya wastewater plant in Baghdad, Iraq, and 40 control subjects were included. The levels of lead and cadmium were measured in blood of the study subjects using the atomic absorption spectroscopy (AAS) method. 8-OHdG was analysed using enzyme-linked immunosorbent assay (ELISA) technique. *δ*-ALAD and U*δ*-ALA were estimated using spectrophotometry-based methods. Our work showed that workers had a significantly higher level of lead and cadmium when compared with the control group (*P* < 0.05), yet, still within the World Health Organization permissible limit. The level of both metals was positively associated with duration of work at the plant (*P* < 0.01). The activity of *δ*-ALAD was inversely associated with the lead level, while both U*δ*-ALA and 8-OHdG were positively correlated with the lead level (*P* < 0.05). These three markers lacked any statistically significant association with the cadmium level (*P* > 0.05). To sum up, working at the wastewater treatment plant was associated with a higher blood level of lead and cadmium and their possible health hazard. Health and occupational safety authorities are required to set up tighter regulations and protocols to minimize these hazards and ensure a safe working environment.

## 1. Introduction

Wastewater is a complex mixture of water carrying waste that is drained from residential, commercial, and industrial establishments [1]. Because sources of waste are different, wastewater contains biological hazard materials, relatively large amounts of possible carcinogens and heavy metals [2–4]. Fruits and vegetables irrigated with wastewater or treated with its sludge as a fertilizer include levels of heavy metals such as lead and cadmium that exceeded the permissible limits established by the World Health Organization (WHO) and the Chinese State Environmental Protection Administration (SEPA) [5, 6].

Heavy metals can be defined as any metallic elements with relatively high density when compared to water; these metals are toxic or poisonous even at minute concentrations [7]. Toxic inorganic heavy metals such as lead (Pb) and cadmium (Cd) are found in the Earth's crust and released to the environment due to human industrial activities such as metal plating, mining, battery production, smelting operations, and paint production [8].

Lead and cadmium are pollutants and are not, normally, present in the human body and have no reported beneficial biochemical activity [9]. Heavy metals are characterised by high solubility in the aqueous solutions and quick absorption by all living organisms [10]. These two features allow these metals to be present in almost all tissues and organs [10]. The concentration of these metals in the human body (bioavailability) is a function of physical (temperature, adsorption, and sequestration) and biochemical factors, which play a role in complexation kinetics, speciation, and lipid solubility [8].

Lead compounds were classified by the International Agency for Research on Cancer (IARC) as Group 2A carcinogens (likely to cause human cancer) and as probable human carcinogens by the US Environmental Protection Agency (EPA) [11, 12]. Lead gets access to the body through either ingestion, inhalation of arousal, or contact with skin of lead-containg materials [13]. Exposure to lead causes genotoxic effects such as chromosome aberration, mutation, DNA breakage, and DNA synthesis inhibition [14]. Markers of nuclear and mitochondrial DNA damage due to lead and cadmium toxicity include 8-hydroxyguanine (8-oxo-7,8-dihydroguanine), 8-hydroxy-2′-deoxyguanosine (8-OHdG), or 8-oxo-7,8-dihydro-2′-deoxyguanosine (8-oxodG) [15]. 8-OHdG is the main form of DNA damage induced by free radicals, which is used as a measurable biomarker for DNA oxidative stress and carcinogenesis [15].

The deleterious effect of lead extends to some enzymes such as delta-aminolevulinic dehydratase (*δ*-ALAD), and this enzyme activity inhibition is a sensitive marker of lead toxicity [12, 16]. Inhibition of *δ*-ALAD impairs the condensation of two molecules of delta-aminolevulinic acid to form porphobilinogen (PBG) in the pathway for haemoglobin biosysnthesis, which leads to the accumulation of ALA in blood with subsequent overflow excretion in urine [13, 17]. *δ*-ALA accumulation stimulates the production of reactive oxygen species (ROS) that culminate in oxidative stress, with generation of 4,5-dioxovaleric acid as the ALA final oxidation product [9, 18]. 4,5-Dioxovaleric acid is an effective alkylating agent of the quinine moieties within both nucleoside and isolated DNA [18]. Alkylation increases the level of 8-oxo-7,8-dihydro-2-deoxyguanosine and 5-hydroxyl-2-deoxycytidine [18].

Cadmium poisoning is a worldwide health concern, which was reported by different countries, and its carcinogenic effects are well recognised [19]. Similar to lead, cadmium (Cd) was classified by the IARC as Group 1 human carcinogen and was considered Group 2a human carcinogen by the EPA [16]. Cadmium enters the human body through different contaminated elements such as air, water, soil, and food [20]. The spectrum of cadmium toxicity consequences ranges from cancer to toxicity of skeletal, urinary, reproductive, cardiovascular, nervous, and respiratory systems [20]. Cadmium delivers its toxic and carcinogenic effects by competitive binding to areas in enzymes, proteins, and DNA (specifically with a zinc finger motif), which are vital for gene regulation, enzyme activity, or maintenance of genomic stability [21]. It also impairs mitochondrial electron transport and stimulates cellular reactive oxygen species generation [22].

The serious and wide-spread toxic effects of lead and cadmium have been a hotspot for research studies in different disease settings and countries, and many meta-analyses were published about the studies in this field [23–27].

Wastewater, as explained earlier, includes high concentration of heavy metals, namely, lead and cadmium, and thus, workers at wastewater might be at a greater risk of the exposure to these metals. However, no study was done, at least in Iraq, to analyse the level of lead and cadmium in blood and their genotoxic effects of the workers at wastewater treatment plants. In this work, we will examine the occupational effect on the level of lead and cadmium and their possible effect on the DNA by measurement of 8-OHdG. We also tried to identify the mechanism by which these metals exert their genotoxic effect by estimation of *δ*-ALAD and U*δ*-ALA.

## 2. Subjects, Materials, and Methods

### 2.1. Al-Rustumiya Wastewater Treatment Plant: An Overview

The plant was built in 1963 and is located on the south-east side of Baghdad, the capital of Iraq, and treats wastewater that is drained from different parts of the capital. The drained area has a population of nearly 3 million and occupied by residential areas, industrial states, and 28 hospitals [28]. Treated water then re-enters the river of Diyala [28]. The plant has seen many expansions to cope with the increase in the population and industrial activities [28].

### 2.2. Study Subjects

This case-control study contained two groups. The first consisted of 79 workers at the Al-Rustumiya wastewater treatment plant, sixty males and 19 females, with age range of 18–65 years (mean and SD 37.06 ± 12.18). The second is the control group of 40 healthy volunteers, who are not working at similar occupations, with matching sex, age, and body mass index (BMI); both groups were from Baghdad. The study was done over a 6-month period from the 1^st^ of October 2016 till the 31^st^ of March 2017.

All subjects were informed about the purposes, benefits, and risks of the study, as well as their right to withdraw at any time in accordance with the Declaration of Helsinki (1964). The study was approved by Al-Nahrain University, Medical College Ethics Committee.

Medical, surgical, and drug intake and occupational history were taken. We excluded those who work at lead- and cadmium-related fields such as petrol stations, battery industry, lead-based painting, pottery, plating, soldering, welding, and printing of books. Cigarette smoking is marked by the increase of blood cadmium; thus, smokers were also exempted from our study [21].

### 2.3. Materials

The laboratory investigations were performed at the Department of Chemistry and Biochemistry at the College of Medicine, Al-Nahrain University, Baghdad, Iraq. They included measurement of blood lead (Pb), cadmium (Cd), 8-OHdG and *δ*-ALAD levels, and estimation of urine concentration of *δ*-ALA.

Morning peripheral venous blood samples were collected between 9:00 AM and 11:00 AM. Serum was obtained from five millilitres (ml) of peripheral venous blood that was collected in a plain tube and centrifuged using the Medifuge™ machine (Thermo Fisher Scientific™, USA) for 15 minutes (min) at 3000 rpm (755 ×g) following clotting. Serum samples were then frozen at −20°C in small aliquots until further analysis within 6-month period for 8-DHoG, while heparinised whole blood was used to measure the concentration of Pb, Cd, and erythrocyte *δ*-ALAD enzyme activity.

Random urine samples were used to measure *δ*-ALA (U-ALA) instead of 24-hour urine sample collection to avoid possible errors from the inadequate and improper collection.

Throughout the study, strict precautions were exercised to prevent contamination of the samples, test reagents, and equipment with lead and cadmium according to the Clinical and Laboratory Standards Institute criteria [29].

### 2.4. Methods

#### 2.4.1. Determination of Lead and Cadmium Concentration in Whole Blood

Shimadzu model AA-6300G® atomic absorption spectrometer (Kyoto, Japan) with the GFA-EX7i graphite furnace atomizer (to vaporize the sample) was used to measure lead and cadmium as described by Subramanian and Meranger [30]. In brief, heparinised whole blood samples and standards were diluted at a ratio of 1 : 10 with distilled water, and whole blood was then homogenised with 25 ml of 10% triton X-100 (Sigma-Aldrich® Company, Germany). 5 ml of 20% ammonium dihydrogen phosphate (Merck-Darmstadt®, Germany), with 1 ml of concentrated nitric acid (BDH Chemicals®, UK) was then added to the sample, and the volume was titrated to 500 ml with distilled water. The measurement was done at 283 nm and 228 nm for lead and cadmium, respectively. The results were presented as *μ*g/dl.

#### 2.4.2. Estimation of the Activity of Delta-Aminolevulinic Acid Dehydratase (*δ*-ALAD) in Erythrocytes

The concentration of *δ*-ALAD in whole blood was measured using Burch and Siegel method as previously described [31]. Shortly, 0.2 ml of heparinised whole blood was homogenised with 1.3 ml of Triton X-100 (Sigma-Aldrich® Company, Germany) and then 1 ml of buffered ALA substrate was added and mixed well. 1 ml of the resultant mixture was utilised as blank. The remaining amount was then incubated at stable pH (7.0) for one hour at 38°C followed by the addition of 1 ml of the TCA reagent and then mixed well and centrifuged. 1 ml of the supernatant was used as the sample. 1 ml of modified Ehrlich's reagent (Sigma-Aldrich® Company, Germany) was added to the already prepared 1 ml of blank and sample tubes and allowed to react. The tubes were allowed to develop colour for 13 minutes before measuring absorbance at 555 nm using a spectrophotometer (Biotech-UV2601®-UK). The results are expressed as mmol/ml of erythrocyte/hr.

#### 2.4.3. Determination of Urinary *δ*-ALA Concentration

The measurement of U*δ*-ALA was performed according to the method described by Wada et al. [32]. This method depends on the development of reddish colour when chloroform is added to urine containing high amount of *δ*-ALA, while faint yellow or faint red colour represents normal amounts of *δ*-ALA in urine. In brief, we added 2 ml of 20% acetic acid solution to a tube containing 2 ml of urine followed by loading 8 ml of *n*-butanol to the mixture and homogenised it well using the vortex shaker (Vortex-Genie®, Scientific Industries, USA). Two test tubes were then used; each tube contained 0.5 ml of the mixture aqueous part, one was used as a blank after adding 1.5 ml of sodium phosphate buffer only, while the other (sample tube) was loaded with 1.5 ml of sodium phosphate buffer containing ethyl acetoacetate. Following ten minutes of incubation of both tubes in boiling water, they were allowed to cool down. Then, 2 ml of Ehrlich's reagent (Sigma-Aldrich® Company, Germany) was mixed. The samples were allowed to settle down for another ten minutes; after that, 4 ml of chloroform was added and mixed well with the vortex shaker. After 5 to 30 min, the absorbance of the chloroform phase was read at 556 nm by the spectrophotometer (Biotech-UV2601®-UK) against the blank. The concentration in urine was shown as *μ*mol/l.

#### 2.4.4. Measurement of 8-Hydroxyl-2-deoxyguanosine (8-OHdG) Concentration

The level of 8-OHdG was measured depending on enzyme-linked immunosorbent assay (ELISA) technology. The measurement kit was supplied by Cayman Chemical® (USA, Michigan 48108, Kit no. 89320) and used according to the manufacturer's instructions. Biotek ELx800® ELISA system (BioTek Instruments, Inc., USA) was used for reading absorbance at 450 nm, and the result was expressed as ng/ml.

### 2.5. Statistical Analysis

GraphPad Prism 8® software (San Diego, CA, USA) was utilised to generate figures and perform statistical analysis. Unpaired Student's *t*-test was used to compare the variables of the two groups. Pearson correlation was applied to study the correlations between various variables depending on the correlation coefficient and *P* value. *P* values <0.05 were considered statistically significant. Test for normality was done using the Kolmogorov–Smirnov test of normality.

## 3. Results

### 3.1. Study Population

There were no statistically significant differences between the worker and control groups with regard to age, sex, body mass index, and haemoglobin (*P* > 0.05, Table 1). The clinicodemographic characteristics of our worker group showed that the age range was 18–65 years with male dominance (80%). Numerical details are shown in Table 1.

### 3.2. Level of Lead and Other Variables and Their Association

**Figure 1:**
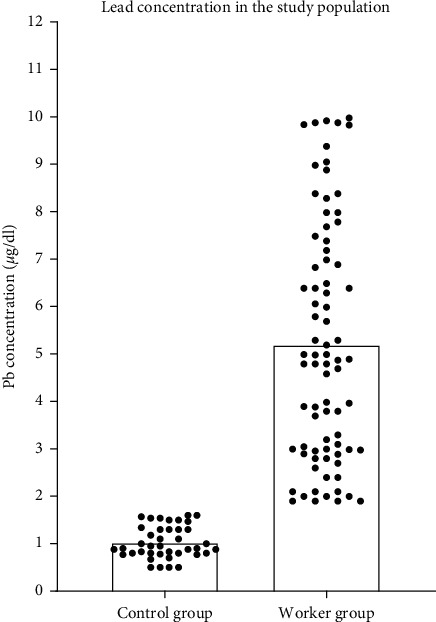
The concentration of lead in the wastewater treatment plant workers is almost five times higher than the amount found in the control group (*P* < 0.0001).

The mean blood level of lead (Pb) of the worker group (5.2 *μ*g/dl) was significantly higher (*P* < 0.0001) when compared with the mean level of the control one (1.03 *μ*g/dl), as can be seen in Figure 1. Gender has no influence on the level of lead within the same group (*P* > 0.05).

**Figure 2:**
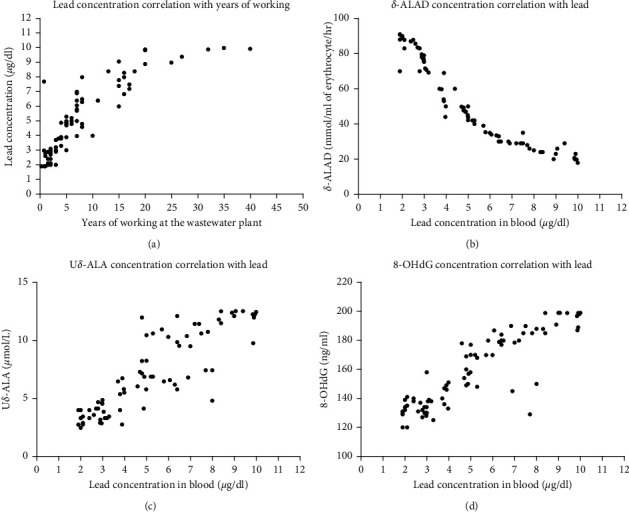
The correlation between years of working with the lead concentration and other variables' (*δ*-ALAD, U*δ*-ALA, and 8-OHdG) level with lead concentration. (a) It is clear that the level of lead rises with the increase in the duration of working at the wastewater treatment plant; a statistically significant positive correlation is seen. (b) Inverse relation between lead level and erythrocyte *δ*-ALAD activity. (c, d) A pattern of positive correlation between U*δ*-ALA and 8-OHdG with lead concentration, respectively.

The level of lead was positively associated with duration of working (correlation coefficient, *r*: 0.86, 95% confidence interval, CI: 0.79 to 0.91, *P* < 0.0001), as clarified in Figure 2(a). As shown in Figure 2(b), it was interesting to find that the ALAD level showed inverse correlation with the lead level (*r*: −0.99, 95% CI: −0.99 to −0.97, *P* < 0.0001). There was a significant positive correlation between the level of urinary ALA and lead (*r*: 0.88, 95% CI: 0.82 to 0.92, *P* < 0.0001), as depicted in Figure 2(c).  Figure 2(d) shows that there is a positive significant correlation between 8-DHoG level and lead concentration (*r*: 0.88, 95% CI: 0.82 to 0.92, *P* < 0.0001).

### 3.3. Level of Cadmium and Other Variables and Their Association

**Figure 3:**
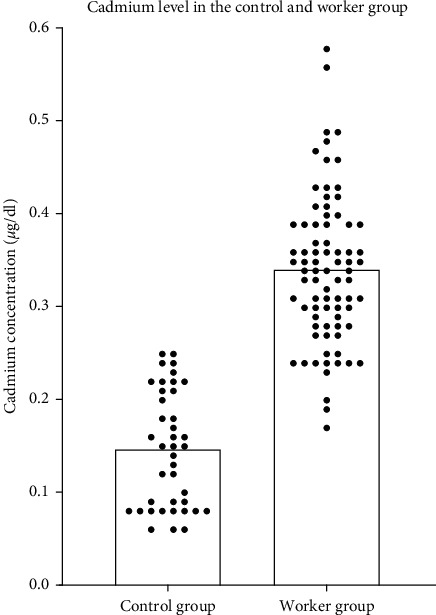
The mean concentration of cadmium in the wastewater treatment plant workers is almost threefold higher than the level found in the control group.

There was significantly higher concentration (*P* < 0.0001) of cadmium in blood of the worker group (mean, 0.34 *μ*g/dl) in comparison with the control group (mean, 0.16 *μ*g/dl), as shown in Figure 3.

**Figure 4:**
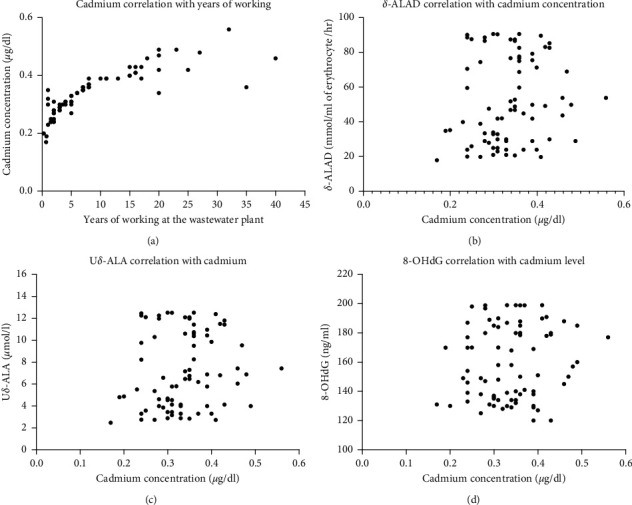
The correlation between cadmium and years of working and other variables' (*δ*-ALAD, U*δ*-ALA, and 8-OHdG) level with cadmium concentration. (a) Concentration of cadmium increases progressively with the increase in the duration of working at the wastewater treatment plant; a statistically significant positive correlation is seen. (b) No correlation between ALAD level and cadmium. (c, d) Absence of any correlation between U*δ*-ALA and 8-OHdG with cadmium, respectively.

We found that the level of cadmium in blood is positively associated with the length of working at the plant (Figure 4(a)) (*r*: 0.81, 95% CI: 0.73 to 0.92, *P* < 0.0001).

The level of *δ*-ALAD is not associated with cadmium concentration (*r*: 0.13, 95% CI: −0.09 to 0.34, *P*=0.242), as visually depicted in Figure 4(b). Another variable we measured was urinary *δ*-aminolevulinic acid (U*δ*-ALA), which showed no correlation with cadmium concentration, as shown in Figure 4(c) and confirmed statistically (*r*: 0.14, 95% CI: −0.087 to 0.35, *P*=0.23). The last variable we assessed was DNA damage marker 8-OHdG which showed no association with the cadmium level (*r*: 0.14, 95% CI: −0.05 to 0.35, *P*=0.23) (Figure 4(d)).

## 4. Discussion

It is generally accepted that lead and cadmium are present in high concentration in wastewater, and these metals have high rate of absorption and long half-life once they get access to the body through multiple routes [2–4, 10]. These observations raise concerns as to whether workers at wastewater treatment plants are at an increased risk of exposure to lead and cadmium. Therefore, we wanted to first measure the level of lead and cadmium in blood of workers and, second, to investigate the genotoxic effect of these elements on workers' DNA. In this work, an effort was, also, made to identify the mechanisms by which this, possible, genotoxic effect occurs by measuring *δ*-ALAD and U*δ*-ALA, and ultimately, we evaluated the level of the DNA damage utilising 8-OHdG as the representative marker.

We found that the level of lead was higher in the worker group compared with the control group. The mean level of lead in the worker group (5.2 *μ*g/dl) was within WHO permissible blood lead concentrations of <10 *μ*g/dl, which progressively increased with the duration of working [33]. This progressive increase can be attributed to lead's nature as a fat-soluble metal with long half-life that is readily absorbed when gets access to the body through ingestion, skin, and inhalation and accumulates in various organs [8, 13, 20]. The last two routes are most reasonable entry routes in our study setting similar to what was described in Sewer Worker's Syndrome [34]. Workers in the wastewater treatment plant and farmers using sludge as a fertilizer had higher level of lead [34]. Furthermore, higher levels were seen in workers in occupations that expose the worker to heavy metals such as mining, melting, and battery industry [35–37]. It might even affect people living in the vicinity of these industries [38]. A paper from Iran has shown that the resident of Tehran, the air-polluted capital of Iran, had level of lead and cadmium that was higher than WHO allowed limits [39].

Our results support the well-recognised finding that lead can inhibit the enzyme ALA dehydratase activity and, expectedly, will increase ALA in blood and consequently its excretion in urine [12, 13, 16, 17]. Although some of the workers have had the same period of work at the plant and had the same levels of lead, their levels of U*δ*-ALA were different due to the presence of single-nucleotide polymorphisms in the *δ*-ALAD gene [30]. Despite *δ*-ALAD activity inhibition, none of our study subjects had anaemia which develops when blood lead level reaches above 50 *μ*g/dl [10].

ALA is known to be carcinogenic through production of 8-hydroxy-2′-deoxyguanosine (8-OHdG), 8-hydroxyguanine (8-oxo-7,8-dihydroguanine), and 8-oxo-7,8-dihydro-2′-deoxyguanosine (8-oxodG) [15]. Mechanistically, ALA generates reactive oxygen species which cause DNA base modification [15]. Similar to our finding, other studies observed a positive relation between 8-OHdG and 8-oxodG with lead [40–42]. Additionally, lead can induce DNA damage, on its own, without ALA mediation [14].

Cadmium toxicity is a global public health problem due to occupational and nonoccupational sources of exposure [13]. In this work, mean cadmium level was higher in the worker group in comparison with the control one, and the level increases with work duration, yet, still within the WHO acceptable level (<0.5 *μ*g/dL) [21]. Workers at smelters and zinc ore refinery had much higher level of cadmium that is greater than the WHO limit, and the level progressively increases with the work period [43, 44].

Cadmium did not show the altered biochemical cascade, seen with lead, i.e., reduction in blood *δ*-ALAD and increase in U*δ*-ALA and 8-OHdG. It was previously shown that cadmium stimulates enzyme ALAD and antagonises the inhibitory effect of lead [45, 46]. To add insult to the injury, another study contradicts the above finding and claims that cadmium inhibits ALAD activity and increases blood and urine *δ*-ALA [47]. Indeed, it is possible that our relatively small sample size might have led to bias in results. The concentration and activity of any substance, including *δ*-ALAD and 8-OHdG, are not the functions of one factor only; rate of production and removal and inhibiting and activating factors are also involved. These depend on the tested tissue type and cellular milieu and, yet to be identified, other factors.

The absence of correlation between ALAD and 8-OHdG with cadmium does not mean the latter is not carcinogenic in our study setting. The well-recognised carcinogenic effect of cadmium is not necessarily delivered through ALA only; other mechanisms are involved such as genomic instability, epigenetic changes, and its estrogenic activity [20].

We have to keep in mind that lead and cadmium in blood represent a very small percentage (2%) of their total amount that is present in the body [13]. Thus, it is considered misleadingly low and does not reflect the actual amount present in the body; it accumulates first in the tissue such as the bone and kidney and then the level reaches the plateau in blood [13]. Additionally, the WHO states that neurological symptoms can occur at lead level of about 5 *μ*g/dl, especially in children [33]. More sophisticated laboratory methods, such as urine provocation test, are needed to estimate the actual amount of cadmium in the body [13].

Our study is limited by its small number of cases; moreover, we measured only the blood level of the heavy metals which does not reflect the total body burden. Other methods to measure DNA damage or gene expression studies have not been used which can give better idea about the extent of genotoxicity.

## 5. Conclusions

To sum it up, both lead and cadmium are higher in the worker group relative to the control group. Lead has shown a greater association with DNA damage, using 8-OHdG as a marker, than cadmium. These observations should stimulate occupational health authorities and decision makers to search for strategies to reduce the exposure of the workers to the heavy metals and ultimately their subsequent health hazard. The presence of permissible limit should not be considered safe and assuring since the tissue level is not known and might be in the toxicity level. More complex laboratory methods should be used to estimate the actual heavy metal burden on the body. It is important to periodically monitor the level of these metals in blood and body and screen for their associated diseases such as various cancer and kidney diseases. Occupational health authorities should define a maximum work period at wastewater plants when workers have to relocate their jobs to places with less heavy metal exposure. This is especially important for those who exceeded the WHO permissible limit.

## Figures and Tables

**Table 1:** Clinicodemographic characteristics of the study population, both the worker group and the control one. Both groups have no differences of statistical significance with regard to the parameters; SD is standard deviation.

Parameter	Control group	Worker group	*P* value
Age (mean ± SD) (years)	36.87 ± 11.7 (24–60 years)	37.06 ± 14.18 (18–65 years)	>0.05

Sex			>0.05
Male	80%	75%	
Female	20%	25%	

BMI (mean ± SD) (kg/m)	25.73 ± 2.2	27.09 ± 4.96	>0.05
Duration in work/years			
0–10 years		57 (72.2%)	
11–20 years		17 (21.5%)	
21–30 years		2 (2.5%)	
31–40 years		3 (3.8%)	

Haemoglobin, Hb (g/dl)			
Mean ± SD	13.5 ± 1.0	13.9 ± 1.2	>0.05

## Data Availability

The data that support the findings of this study are available from the corresponding author (Salih Ibrahem) upon request.
